# Evaluation of Knowledge and Practice of Hairdressers in Women’s Beauty Salons in Isfahan About Hepatitis B, Hepatitis C, and AIDS in 2010 and 2011

**DOI:** 10.5812/hepatmon.6215

**Published:** 2013-03-06

**Authors:** Behrooz Ataei, Kiana Shirani, Seyed Moayed Alavian, Mehdi Ataie

**Affiliations:** 1 Infectious Diseases and Tropical Medicine Research Center, Isfahan University of Medical Sciences, Isfahan, IR Iran; 2 Nasocomial Infection Research Center, Isfahan University of Medical Sciences, Isfahan, IR Iran; 3 Baqiyatallah Research Center for Gastroenterology and Liver Diseases, Baqiyatallah University of Medical Sciences and Tehran Hepatitis Center, Tehran, IR Iran; 4 Tehran Hepatitis Center, Tehran, IR Iran; 5 Young Researchers Club, Najafabad Branch, Islamic Azad University, Isfahan, IR Iran

**Keywords:** Hepatitis B Virus, Hepatitis C, Acquired Immunodeficiency Syndrome, Knowledge, Women, Iran

## Abstract

**Background:**

Blood-borne viruses such as hepatitis B, hepatitis C, and human immunodeficiency virus (HIV) have some common epidemiological characteristics, and have infected millions of people throughout the world. Patients infected by acute hepatitis or HIV infections may not be aware of the disease, and thereby cause transmission to others. During haircut, shave, or pedicure, barbers may accidentally expose to their clients’ blood, transmit their own infection to them, or transmit the infection from one client to another. Thus the beauty salon staff has a potential role in expansion of infections.

**Objectives:**

As being barbers and barbering are risk factors to some infectious diseases, determining the role of knowledge and awareness of barbers and hairdressers about topics related to AIDS, and hepatitis B and C is important.

**Patients and Methods:**

This cross-sectional descriptive study was performed in 2010-2011 on 281 women’s beauty salons located in eleven urban districts of Isfahan town. A multistage cluster sampling was performed and knowledge assessment questionnaire accompanied by practice checklist regarding hepatitis B and C, and AIDS were completed by trained interviewers. Knowledge and practice scores were determined in 0-20 and 0-10 scales, respectively. The content validity of questionnaire was confirmed by three expert opinions and the test-retest reliability of the questionnaire was determined to be 0.83 in a pilot study on 30 participants. Data were analyzed using Pearson’s correlation coefficient and one-way ANOVA test by SPSS software, version 18.

**Results:**

In the study, 281 hairdressers participated. There was a statistically significant relationship between education level and knowledge score of hairdressers (P < 0.001). We found a statistically significant relationship between knowledge level and job history of hairdressers according to the Pearson’s correlation coefficient (P = 0.004). The results did not show any statistically significant relationship between education level and practice scores (P = 0.5). Furthermore, the job history of hairdressers and their practice score did not show a significant relationship (P = 0.77).

**Conclusions:**

We obtained promising results about the knowledge and practice levels of staffs of women’s beauty salons in Isfahan about AIDS, and hepatitis B and C. The target group exhibited an intermediate level of knowledge about the diseases because of important role of barbers in virus transmission; we should provide the best control, evaluation, and continuous teaching programs.

## 1. Background

Blood-borne viruses such as hepatitis B, hepatitis C, and human immunodeficiency virus (HIV) have some common epidemiological characteristics, and have infected millions of people throughout the world (1). Prevalence of these diseases in barbershops in countries such as Ethiopia, Pakistan, and Bangladesh has been reported to be 34 to 49 % (2, 3). prevalence of hepatitis B is higher in Asia, Africa, South America, Middle East, Pacific Islands, and East Europe; such rate in Sub-Saharan and Southeast Asian countries and Alaska is high (8-20 %) and in medium level in Mediterranean countries, Japan, Central Asia, Middle East, and South America. Moreover, prevalence rate is low (1-2 %) in the USA, Canada, West Europe, Australia, and New Zealand. The Center for Disease Control and Prevention (CDC) estimates that almost 1.25 million people are infected by hepatitis B, and approximately 5 % of the world populations are carriers of hepatitis B. prevalence rate of hepatitis B carriers in different parts of Iran has been reported to be 0-3.9 %. Most patients with acute hepatitis B are completely cured within 4-6 weeks, and only a small number of the cases (almost 1 per 300 cases) may end in hepatic failure and death. Moreover, 1/20 of the cases progress into chronic hepatitis. In general, the major complications of chronic hepatitis B are cirrhosis and hepatic cancer (4, 5). With regard to hepatitis C, the World Health Organization (WHO) estimates that 170 million individuals around the world are chronically infected by hepatitis C virus (HCV) and almost 3-4 million new cases of the infection occur annually. In Iran, it seems that prevalence of the infection in general population is less than 1 %. In approximately 80 % of acute hepatitis C patients, the infection develops into chronic hepatitis C, among which 10-20 % leads to cirrhosis and 1-1.5 % of them end in hepatic cancer within 20-30 years (6, 7). Currently, hepatitis C is the major cause of post-transfusion hepatitis. HCV is a main reason for hepatitis in Intravenous drug users (IVDU). Moreover, the virus is responsible for at least 50 % of the cases of community acquired sporadic hepatitis, and in many cases the route of virus transmission is unknown (8, 9). So far, appropriate treatments for these infections have not been introduced. Furthermore, more than 50 % of the infections are subclinical, and are not usually reported. HIV infected individuals also may remain asymptomatic for years. Thus, patients with acute hepatitis or HIV infection may not be aware of the infection, and cause transmission to others. These infections are transmitted through body fluids such as saliva, urine, sweat, semen, and vaginal secretions, as well as blood and blood products (10). During haircut, shave, or pedicure, barbers may accidentally expose to their clients’ blood (11) and transmit their own infection to them, or transmit the infection from one client to another. For instance in some areas of Nigeria in barbershops the risk of HIV transmission is high, or in Iran Hepatitis B virus infection in barbers is more than that of normal population (12, 13). The knowledge and awareness of barbers and hairdressers about topics related to AIDS, and hepatitis B and C infections are of great importance and there are some unsafe practices that may lead to infections due to blood-borne viruses (14), for example negligence in using beauty instruments such as needles for tattooing and razors for shaving can make these tools important factors in transmission of the infection from one client to another. Also Potash Alum being used by barbers on facial shaving cuts has definite role in HCV transmission in Pakistani population (15). Thus, if not following the health measures, the beauty salon staff plays a potential role in expansion of infections such as AIDS, and hepatitis B and C. Accordingly and also the fact that no study has been performed in Isfahan to evaluate knowledge and practice of staffs in women’s beauty salons about AIDS, hepatitis B and C, the current study was conducted. In the study, we evaluated the knowledge and practice of staffs in women’s beauty salons in Isfahan about the diseases. The results would be helpful in determination of the knowledge and practice levels of the staffs in different regions of the city, according to the job history and education level. A pattern for educational intervention can then be prepared that would decrease the risk of transmission and expansion of the three infections among the staffs and general population.

## 2. Objectives

This study was performed to evaluate the knowledge level and practice of the women’s beauty salon staffs in Isfahan about hepatitis B, C, and AIDS in 2010-2011.

## 3. Patients and Methods

This cross-sectional descriptive study was performed in 2010-2011 in women’s beauty salons in eleven urban districts of Isfahan town, which were registered in hairdressers’ syndicate and were willing to participate in the study. Those who were not willing to attend the study were excluded. A multistage sampling was performed and 281 female hairdressers were selected. To select the participants, the names and addresses of women’s beauty salons in Isfahan were taken from the syndicate, and proportionate to the number of salons in each district, some salons were selected using the computer-based random number table. Finally, 281 hairdressers were included in the study. Sample size was calculated based on our previously conducted pilot study. The knowledge assessment questionnaire and the practice checklist regarding hepatitis B and C, and AIDS were completed by trained interviewers. The questionnaire included items on the transmission routes, prevention, vaccination, and treatment of the infections. The test-retest reliability of the questionnaire was determined to be 0.83 based on a pilot study on 30 participants. The knowledge scores were determined in a 0-20 scale, and respective knowledge levels ranked as follows: desirable (score 16-20), intermediate (score 11-15), weak (score 6-10), and very weak (score 0-5). The practice score was determined in a 0-10 scale and then ranked as follows: very desirable (score 9-10), desirable (score 7-8), intermediate (score 5-6), inappropriate (score 3-4), and very inappropriate (score 0-2) practice levels. The data were collected in salons by completing a checklist and a questionnaire and analyzed using Pearson’s correlation coefficient and one-way ANOVA test by SPSS software, version 18. The protocol of the study was approved by internal ethics committee of Isfahan University of medical sciences.

## 4. Results

In the study, 281 hairdressers participated. The mean job history of the participants was 5.88 ± 5.39 years, with the maximum and minimum values of 30 years and four months, respectively. With regard to their education level, 40 (14.28 %), 217 (77.14 %), and 24 (8.57 %) hairdressers had the education level below-high-school diploma, high school diploma, and academic, respectively. (P value = 0.01) The mean knowledge score obtained by the participants was 14.05 ± 2.25 (in a 0-20 scale), with the minimum and maximum of eight and 19, respectively. In this respect, 86 (30.6 %), 162 (57.6 %), and 33 (11.7 %) hairdressers had desirable, intermediate, and weak knowledge level. The mean knowledge level about AIDS, and hepatitis B and C with regard to the education level of the participants is provided in Table 1 and the frequency distribution of the responses to questioner is presented in Table 2.

**Table 1:** Frequency Distribution of Knowledge of Isfahan Women’s Hairdressers About AIDS, and Hepatitis B and C With Regard to Their Education Level

	Frequency	Mean ± SD	F value	*P*value
**Hepatitis B**				
Below high school diploma	40	9.05 ± 2.51		
High school diploma	241	12.08 ± 1.97		
Total	281	11.34 ± 2.21	26.13	< 0.0001
**Hepatitis C**				
Below high school diploma	40	8.85 ± 2.33		
High school diploma	241	12.05 ± 2.29		
Total	281	11.003 ± 2.31	24.75	< 0.0001
**AIDS**				
Below high school diploma	40	8.12 ± 1.96		
High school diploma	241	11.40 ± 1.99		
Total	281	10.15 ± 2.13	27.58	< 0.0001

According to the one-way ANOVA results, there was a statistically significant relationship between the education level and knowledge score of hairdressers (P < 0.001). We found a statistically significant relationship between knowledge level and job history of hairdressers according to the Pearson’s correlation coefficient (P = 0.004). The mean score obtained by participants in the practice was 4.7 ± 1.47, with the minimum and maximum scores of 0 and eight, respectively. The frequency distribution of Items of practice is presented in Table 3. According to the results, the practice of none of participants lied within the very desirable level. Moreover, the practice of 14 (10 %), 64 (45.7 %), 52 (37.1 %), and 10 (7.1 %) hairdressers were desirable, intermediate, inappropriate, and very inappropriate, respectively. In Tabulation, the mean and standard deviation of practice scores obtained by the hairdressers according to their education level is provided. The results of one-way ANOVA did not show any statistically significant relationship among the three education levels (P = 0.5). Furthermore, the job history of hairdressers and their practice score did not have a significant relationship (P = 0.77).

**Table 2:** Frequency Distribution of the Responses of Women’s Hairdressers in Isfahan about Aids, And Hepatitis B and C

Item	No, No. (%)	Yes, No. (%)
**Can AIDS be transmitted through kissing?**	86 (30.7)	195 (69.3)
**Can AIDS, and hepatitis B and C be transmitted through sharing toilet seats?**	107 (38.2)	1784 (61.8)
**Can AIDS, and hepatitis B and C be transmitted through insect bit?**	178 (63.2)	103 (36.8)
**Can AIDS, and hepatitis B and C be transmitted from mother to child?**	9 (3.2)	272 (96.8)
**Can interfamilial transmission of AIDS, and hepatitis B and C occur?**	171 (60.7)	110 (39.3)
**Can AIDS, and hepatitis B and C be transmitted through sexual intercourse?**	4 (1.4)	277 (98.6)
**Can AIDS, and hepatitis B and C be transmitted through talking with the infected individual?**	257 (91.4)	24 (8.6)
**Are some specific types of contraceptive methods effective in preventing transmission of hepatitis B and C, and HIV to the partner?**	67 (23.9)	214 (76.1)
**Can AIDS, and hepatitis B and C be transmitted through nail clippers?**	109 (38.9)	172 (61.1)
**Can AIDS, and hepatitis B and C be transmitted through embracing and hand shaking?**	247 (87.9)	34 (12.1)
**Can AIDS, and hepatitis B and C be transmitted through using the unwashed dishes and spoons of the patients?**	157 (55.7)	124 (44.3)
**Should AIDS, and hepatitis B and C patients be completely isolated in the family?**	74 (26.4)	207 (73.6)
**Can AIDS, and hepatitis B and C be transmitted through sharing a towel?**	71 (25.4)	210 (74.6)
**Is there any vaccine for prevention of hepatitis B?**	50 (17.9)	231 (82.1)
**Can AIDS, and hepatitis B and C be transmitted through sharing shaving machines and razors?**	20 (7.1)	261 (92.9)
**Is hepatitis B an incurable disease?**	149 (52.9)	132 (47.1)
**Is there any vaccine for prevention of hepatitis C?**	183 (65)	98 (35)
**Is there any definite treatment for hepatitis C?**	112 (40)	169 (60)
**Is there any vaccine for prevention of AIDS?**	232 (82.5)	49 (17.5)
**Is there any definite treatment for AIDS?**	268 (95.4)	13 (4.6)

**Table 3:** Frequency Distribution of Items of Practice in Isfahan Hairdressers About AIDS, and Hepatitis B and C

Items	No, No. (%)	Yes No. (%)
Is there alcohol burner in the beauty salon?	83 (59.3)	57 (40.7)
Is alcohol burner used for disinfection of instruments?	118 (84.3)	22 (15.7)
Are hair roller clips disinfected in boiling water after each use?	133 (95)	7 (5)
Do all clients have their personal cosmetic suit?	107 (76.4)	3 (23.6)
Do hairdressers use disposable razors for clients?	8 (5.7)	132 (94.3)
Are threading strings changed for each client?	4 (2.9)	136 (97.1)
Are manicure and pedicure rasps disinfected in boiling water after each use?	132 (94.3)	8 (5.7)
Are tattoo instruments disposable?	66 (47.1)	74 (52.9)
Do they use fresh towels for each client?	38 (27.1)	102 (72.9)
Do beauticians take care of not to cause skin injury for the clients by razors, tweezers, and rasps?	53 (37.9)	87 (62.1)

**Tabulation:** Mean and Standard Deviation of Practice Scores of Isfahan Women’s Hairdressers About AIDS, and Hepatitis B and C According to Their Education Level a

	Mean ± SD
**Below high school diploma**	4.87 ± 1.82
**High school diploma**	4.71 ± 1.46
**Academic**	4.2 ± 0.78
**Total**	4.7 ± 1.47

^a^P value = 0.5

## 5. Discussion 

The general aim of this study was to evaluate the knowledge and practice levels of staffs of women’s beauty salons in Isfahan about the transmission routes and prevention of AIDS, and hepatitis B and C in 2010-2011. These infections are transmitted through body fluids, such as blood (10). During haircut, shave, or pedicure, barbers may accidentally expose to their clients’ blood (11); however, prevalence of HBsAg positive and anti-HCVAb are similar to general population (16, 17). Moreover, we analyzed the relationship between knowledge and practice levels in one hand, and job history, education level, and city district in which the staffs work, in other hand. Although hairdressers include a small portion of Isfahan citizens, they belong to the young and dynamic class of the community. Therefore, they are considered as one of the most important groups with regard to their knowledge and practice about transmittable diseases. This study revealed the need to improvement of specific health messages in media campaigns carried out to general population, diffusing more appropriate educational materials for salons, and organizing obligatory update courses for hairdressing sector (18). In this study the mean job history of participants was 5.88 ± 5.39 years. If we consider 25 years as minimum job history required for retirement in Iran, it is necessary to improve knowledge and practice levels of target population about these three diseases.

With respect to the education level, 14.2 %, 77.1 %, and 8.6 % obtained below-high-school diploma, high school diploma, and academic education level, respectively. In Iran, high school diploma is considered as the end of non-academic education period, therefore it seems that the participants were in a good education level. The mean score obtained on knowledge about the three diseases was 14.05 ± 2.25. Thus, the target group had an intermediate level of knowledge about the diseases, which was not unexpected with regard to education level, job history, and particularly the type of their job. In a study carried out in 2005 on 240 soldiers who entered a military training center in southeast of Iran for passing preliminary military trainings, it was reported that their mean knowledge level was intermediate (19). In another study carried out in Vietnam among 345 men in 2005 on their knowledge level about hepatitis B, only 46 % knew that the virus is not transmitted through foods (19). In our study, among 240 hairdressers, only 47.5 % knew that common eating tools and food are not the transmission routes of HIV. Therefore, it can be concluded that the knowledge level in our country is almost similar to that in Vietnam in this regard. In a study carried out in Isfahan high schools to determine knowledge level of students about AIDS and its prevention methods, this level was intermediate (20). If we suppose that the future hairdressers of the city roots in these students, tomorrow knowledge level of this profession would be the same. So, we must carry out programs to improve their knowledge level. In a study fulfilled in Kerman about HIV in 2004-2005, the knowledge and attitude of 164 male and female hairdressers of the city about HIV and AIDS were evaluated. The mean knowledge score of the hairdressers was determined to be 11.1 ± 2.9 (21). Their mean job history was 11.7 ± 9.7 years and 47.6 % of the participants had high school diploma. Moreover, according to the responds of hairdressers, 84.1 % of target group employed their own personal shaving and hairdressing tools. The rate in our study was 10.4 %. In the study mentioned above, 66.8 % of participants considered using razors in the barbershops as the most important tool of AIDS transmission. The rate in our study was obtained to be 97.1 %. Also, they reported that the hairdressers’ knowledge level was intermediate, which was not desirable. In a study performed in Yazd on 140 hairdressers, 24 % of target group (hairdressers) and 19 % of control group (the clients) exhibited a good level of knowledge about hepatitis B in the pre-test (22). In a study carried out in Ankara on 96 female hairdressers, it was shown that they had little information about HIV prevalence and route of transmission (23). In another study conducted in Pakistan, the level of awareness among barbers about hepatitis disease and risks of transmission was very low, and their practice of razor reuse that may spread hepatitis disease was very common (24). In recent years, the interest in improving the knowledge about AIDS and other infectious diseases have increased, and people are eager to get more appropriate information about the diseases. Since hepatitis B and C, and AIDS are considered as more critical diseases, the population is more willing to learn more about them, and this is the responsibility of the health system and mass media to promote population’s knowledge level. We obtained promising results about the knowledge and practice levels of the staffs of women’s beauty salons in Isfahan about AIDS, and hepatitis B and C. However, the results should be carefully analyzed. Presence of confounding variables such as unnatural behaviors of hairdressers due to the presence of raters, high number of unregistered hairdressers working in women’s beauty salons of Isfahan, and high number of hairdressers who were not willing to attend the workshop without mentioning any particular reason are the items which should be considered in interpretation of the results. However, in spite of all limitations and confounding factors, we are optimistic about the findings, but because the important role of barbers in virus transmission, we should provide the best program for control, evaluation and continuous teaching.
